# Special Issue “Polyamines in Aging and Disease”

**DOI:** 10.3390/ijms252211960

**Published:** 2024-11-07

**Authors:** Takeshi Uemura, Yusuke Terui

**Affiliations:** 1 Faculty of Pharmacy and Pharmaceutical Sciences, Josai University, 1-1 Keyakidai, Sakado 350-0295, Japan; 2 Department of Pharmaceutical Sciences, International University of Health and Welfare, 2600-1 Kitakanemaru, Otawara 324-8501, Japan; yterui@iuhw.ac.jp

Polyamines are bioactive amines found in almost all living organisms and are essential for normal cellular functions. The concentration of polyamines in cells is tightly regulated by metabolism: biosynthesis, degradation, and transport. Recent studies have shown that disorders of polyamine metabolism are associated with aging and disease. Age-related reductions in polyamines have been shown to lead to reduced cognitive and physical function. In cancer, on the other hand, high levels of polyamines have been associated with a worse prognosis. As the global population ages, there is a need for technologies to prevent and overcome aging and various diseases and to extend healthy life spans. This Special Issue aims to deepen our knowledge of the relationship between polyamines and aging and disease. This Special Issue invites contributors to publish important work that will clarify the relationship between polyamines and aging and disease and extend the healthy life span by preventing and overcoming various diseases.

Recent updates on the role of polyamines in age-related diseases are reviewed by Jimenez Gutierrez GE et al. [1]. Aging is a major risk factor for degenerative diseases such as Alzheimer’s disease, Parkinson’s disease, osteoarthritis, sarcopenia, and osteoporosis. This review discusses the molecular roles of polyamines in these diseases. They also suggest the potential of polyamines as novel biomarkers for age-related diseases. In addition to degenerative diseases, ovarian aging and the associated decline in fertility are issues that need to be addressed in an aging society. Kang et al. have written a review article on the possibility that polyamines may alleviate ovarian aging through autophagy and oxidative stress and that polyamines may be a target for therapy [2].

In order to achieve the anti-aging effects of polyamines on the human body, it is essential to consider the efficiency of polyamine intake. Ami et al. isolated *Levilactobacillus brevis*, which produces a large amount of putrescine from blue cheese, a food habit, and reported its potential application [3].

In addition to polyamine intake, the suppression of polyamine degradation is also important to inhibit aging. Uemura et al. show that spermine degradation by spermine oxidase (SMOX) plays an important role in cellular senescence [4]. Their data show that cellular senescence can be controlled by SMOX inhibitors.

The oxidation of spermine by SMOX produces acrolein, a highly toxic aldehyde, as a reaction byproduct. Acrolein reacts with various proteins to produce cytotoxic effects. A novel protein mediating acrolein toxicity was identified by Sakamoto et al. [5]. They focused on transient receptor potential ankyrin 1 (TRPA1), which is induced by acrolein-containing cigarette smoke, and found that the high expression of TRPA1 enhanced acrolein toxicity, while the knockdown or antagonist treatment of TRPA1 reduced it, indicating that acrolein toxicity is mediated by this receptor.

The scoping review by Chu et al. discusses the potential of polyamines as biomarkers for oral cancer, periodontal disease, and halitosis [6]. According to their study, polyamines are a convenient and non-invasive potential biomarker for oral status and treatment progress.

Polyamine metabolic imbalances cause a variety of pathologies, including polyaminopathies, such as Snyder–Robinson syndrome, Bachmann–Bupp syndrome, and Deoxyhypusine Synthase Disorder. They are caused by changes in the activity of polyamine-metabolizing enzymes due to genetic factors. Wu and Liu discussed the mechanisms of these diseases based on structural findings of the causative enzymes [7].

Spermidine is a substrate of deoxyhypusine synthase and plays an important role in the activation of the translational regulator eukaryotic initiation factor 5A (eIF5A). Nakanishi and Cleveland provided a comprehensive review of the role of eIF5A in human health [8]. By discussing the aspect of eIF5A in normal and pathological conditions by tissue and cell type, the potential for eIF5A-targeted therapies is demonstrated. Spermidine is not only essential for eIF5A activity, but is also involved in a variety of physiological functions. The authors showed that the localization of SPDS changes with growth [9].

Espinoza-Culupu et al. investigated the anti-inflammatory and antioxidant potential of mygalin, a synthetic analog of spermidine, through in silico and in vitro analysis [10]. Mygalin reduced the LPS-induced expression of iNOS, COX-2, and LPS-induced COX-2 and reduced oxidative stress. These results suggest that mygalin has anti-inflammatory and antioxidant effects.

Liu et al. discuss the benefit of combination strategies targeting tumor cell polyamine homeostasis [11]. The report summarizes the latest findings on the treatment and prevention of cancer by targeting polyamine metabolism and combination therapy.

The contributions in this Special Issue range from the relationship between aging and polyamine metabolism to pathologies caused by disturbances in polyamine metabolic balance and their treatment. We thank all authors and further encourage the development in this research area.
